# 210Protective Effect of Aloe vera Gel against Cisplatin-Induced Testicular Damage, Sperm Alteration and Oxidative Stress in Rats 

**DOI:** 10.22074/IJFS.2020.134691

**Published:** 2021-06-22

**Authors:** Naeem Erfani Majd, Mohammad Reza Tabandeh, Shima Hosseinifar Hosseinifar, Mahin Sadeghi

**Affiliations:** 1 Department of Basic Sciences, Histology Section, Faculty of Veterinary Medicine, Shahid Chamran University of Ahvaz, Ahvaz, Iran; 2 Stem Cell and Transgenic Technology Research Center, Department of Basic Sciences, Faculty of Veterinary Medicine, Shahid Chamran University of Ahvaz, Ahvaz, Iran; 3 Department of Basic Sciences, Division of Biochemistry and Molecular Biology, Faculty of Veterinary Medicine, Shahid Chamran University of Ahvaz, Ahvaz, Iran

**Keywords:** Aloe vera, Cisplatin, Oxidative Stress, Rat, Testis

## Abstract

**Background:**

Cisplatin (CIS) is an effective antineoplastic drug that is used to treat various types of cancers. However, it causes side effects on the male reproductive system. The present study aimed to investigate the possible protective effects of Aloe vera (AL) gel (known as an antioxidant plant) on CIS-induced changes in rat sperm parameters, testicular structure, and oxidative stress markers.

**Materials and Methods:**

In this experimental study, forty-eight adult male rats were divided into 6 groups including: control, CIS, AL, metformin (MET), CIS+AL, and CIS+MET. CIS was used intraperitoneally at a dose of 5 mg/kg on days 7, 14, 21, and 28 of the experiment. AL gel (400 mg/kg per day) and MET (200 mg/kg per day) were administered orally for 35 days (started one week before the beginning of the experiment). Testes weight and dimensions, and morphometrical and histological alterations, activities of antioxidant enzymes including superoxide dismutase (SOD) and glutathione peroxidase (GPx), serum testosterone concentration, lipid peroxidation level, and sperm parameters were examined.

**Results:**

CIS caused a significant decrease (P<0.05) in relative weight and dimension of the testis, germinal epithelium thickness and diameter of seminiferous tubules, the numbers of testicular cells, and spermatogenesis indexes. The malondialdehyde (MDA) levels increased and antioxidant enzymes activities decreased in the CIS group compared to the control group (P<0.05). Additionally, sperm parameters (concentration, viability, motility, and normal morphology), and testosterone levels reduced significantly in CIS-treated rats (P<0.05). Also, CIS induced histopathological damages including disorganization, desquamation, atrophy, and vacuolation in the testis. However, administration of AL gel to CIS-treated rats attenuated the CIS-induced alterations, mitigated testicular oxidative stress and increased testosterone concentration.

**Conclusion:**

The results suggest that AL as a potential antioxidant plant and due to free radicals scavenging activities, has a protective effect against CIS-induced testicular alterations.

## Introduction

Cisplatin [CIS-diaminedichloroplatinum (II)] is one of the most effective anticancer drugs which is used for treatment of a vast variety of human cancers. The anticancer activity of CIS is due to multiple mechanisms such as induction of DNA damage, oxidative stress, and programmed cell death (apoptosis) (1).

Despite the fact that CIS is a useful anticancer drug, it is very toxic and induces several side effects including reproductive toxicity, hepatotoxicity and nephrotoxicity (1, 2). Reproductive toxicity is one of the most common side effects of CIS in treated patients (2-5). CIS causes severe testicular damage which is characterized by apoptosis of germ cell, dysfunction of Leydig cell, testicular steroidogenic disorder and spermatogenic damage (3-6). The precise mechanism of reproductive toxicity induced by CIS is not fully established, however oxidative stress has been known as the major cause of CIS-related testicular dysfunction (5, 6). Hence, several investigators have used antioxidant compounds to reduce reproductive damages caused by CIS (2, 5, 6). For example, olive leaf extract which contains flavonoid and polyphenolic compounds ameliorated CIS-induced testicular oxidative stress in rats (5). Also, fenugreek seed extract reduced oxidative stress and testicular tissue damage induced by CIS and improved spermatogenesis in the rats (6).

*Aloe barbadensis* Miller or AL, is a perennial shrubby plant of the Liliaceae family. It considered an important medicinal herb because of its many medicinal activities including antitumor, antioxidant, anti-allergic, anti-viral, and anti-inflammatory properties. It has been proposed that the antioxidant activity of AL may be a major property of this plant used in the treatment of several diseases. The antioxidant property of AL is due to a large amount of antioxidants substances such as vitamins (A, C, B and E), flavonoids, phenolic compounds, and polysaccharides (7). Several researchers have provided experimental evidence for the reproprotective effect of AL in experimental animals (8-11). These studies have shown that AL can efficiently attenuate the testicular alteration induced by some drugs and heavy metals (8, 9). Other studies reported that AL due to its antioxidant compounds (especially vitamin E) can improve testicular weight, height of the germinal epithelium and diameter of seminiferous tubule, and ameliorate reductions in the number of testicular cells. Also, phenolic and flavonoids contents of AL can be effective in increasing the antioxidant enzymes activity and decreasing lipid peroxidation that can cause extensive damage to cell membranes lipids (8-11). 

It has been found that oxidative stress plays a major role in the pathogenesis of reproductive toxicity induced by CIS. Because of the antioxidant property of AL gel, it was hypothesized that AL may attenuate CIS- mediated gonadotoxicity in rats. Therefore, this study was designed for the first time, to examine possible protective effects of AL gel on gonadotoxicity induced by CIS via evaluation of epididymal sperm parameters, alterations of testicular tissue, testosterone levels, and oxidative/antioxidant markers in the testis of rats. 

## Materials and Methods

### Preparation of *A. vera *gel and analysis of its antioxidant properties

AL gel powder (A. barbadensis) was obtained from Barij Essence Pharmaceutical Co (Kashan, Iran). Total flavonoids content (TFC) was measured by aluminum chloride colorimetric assay (12). The catechin solutions (0-25 μg/mL) were prepared for flavonoid assessment. Aliquots (25 μL) of each AL gel (10 mg in 1 ml distilled water) and standard were mixed with 125 μL distilled water followed by adding 8 μL of 5% sodium nitrate. After 5 minutes, 0.15 ml of 10% aluminum chloride solution was added to 15 μL of that mixture. The absorbance was measured at 517 nm. TFC is expressed as the percentage of catechin equivalents (QE) per 100 g dry weight, and was determined from the standard calibration curve.

Total phenolic content (TPC) of AL gel was estimated using the Folin-Ciocalteu (FC) and aluminum chloride colorimetric assay as described by Im et al. (12). Contents are expressed as the percentage of gallic acid equivalents (GAE) per 100 g dry weight of AL gel.

### Animals and experimental groups

In this experimental study, a total of forty-eight healthy male Wistar rats (180-200 g) were maintained under standard laboratory conditions (12-hour light: 12-hour dark at 22 ± 2 °C) and fed with commercial rat pellets (Pars Animal Feed Co, Tehran, Iran) and water. All experimental assays were approved by the Ethics Committee of Shahid Chamran University of Ahvaz for animal and human experiments (EE/99.3.02.15058/ssu.ac.ir). 

After a quarantine period of 7 days, the rats were divided randomly into 6 groups (n=8) as follows: control group: rats fed with a standard diet and kept in normal conditions. CIS group (CIS): rats received CIS intraperitoneally (i.p) at a dose of 5 mg/kg on days 7, 14, 21, and 28 of the experiment. AL group (AL): AL gel powder was dissolved in distilled water and administered orally at a dose of 400 mg/kg/day for 35 days. MET group (MET): rats received MET (200 mg/kg/day, orally) for 35 days. CIS and AL group (CIS-AL): rats received CIS (i.p) at a dose of 5 mg/kg on days 7, 14, 21, and 28 of the experiment and AL (400 mg/kg/day, orally) for 35 days. CIS and MET group (CIS-MET): rats received CIS (i.p) at a dose of 5 mg/kg on days 7, 14, 21, and 28 of the experiment and MET (200 mg/kg/day, orally) for 35 days. 

The experiment lasted for 35 days (13). The dose of CIS was selected based on a published report (14). The dosing regimen for AL and MET were selected based on reports by Behmanesh et al. (13) and Sahu et al. (15), respectively.

### Sample collection

All rats were anesthetized using ketamine and xylazine (100 mg/kg and 10 mg/kg, respectively), (Alfasan Chemical Co., Woerden-Netherlands) (16). 

The blood samples were collected via cardiac puncture and centrifuged (at 3000 rpm for 10 minutes). Serum samples were separated and then stored at -20˚C for testosterone hormone analysis. Afterward, testes and epididymis were obtained from the abdominal cavity. The weight, dimensions (length and diameter) and volume of testes were measured using a digital scale, a caliper, and water displacement method, respectively (17). The left testis was fixed in a 10% buffered formalin solution for histological analyses and the right testes were stored at -20°C for oxidant/antioxidant assessment. The epididymis tissue samples were used for the analysis of sperm parameters.

### The testicular index

The relative testis weight ratio (%) was calculated using the formula: (absolute weight of the testis/ total body weight)×100 (5). 

### Histological procedures

The formalin-fixed testes were embedded in paraffin blocks, then sectioned (5-μm thickness) by a microtome (Leica RM 2125, Leica Microsystems Nussloch GmbH, Germany). Sections were stained with hematoxylin and eosin (H&E)

### Morphometrical analyses

For this purpose, 100 cross sections of seminiferous were chosen randomly in 5 non-serial sections per animal (10 tubules in the central zone and 10 tubules in the peripheral (sub-capsular) zone of each section). Then, the seminiferous tubule diameter and height of germinal epithelium were measured at ×10 magnification. Also, Sertoli, Leydig, spermatogonia, primary spermatocyte, early spermatid and late spermatid cells were counted in a marked scale (150 µm) at ×40 magnification (18, 19). All measurements were performed under a light microscope (Olympus Optical Co., Japan) using Dino-Lite digital lens (with Dino capture software, FDP2, Taiwan).

Spermiogenesis index (SI) and tubular differentiation index (TDI) were calculated for spermatogenesis assay. SI index was calculated using the following formula:

(Seminiferous tubules contained sperm/seminiferous tubules without sperm)×100.

For TDI index, the percentage of tubules that contained three or more differentiated spermatogenic cells from the type A spermatogonia (i.e. intermediate or type B spermatogonia, spermatocytes, or spermatids) were calculated (18). 

### Analysis of sperm parameters

The cauda epididymis was minced finely in (5 ml) Ham’s F-10 medium and placed at 37°C for 15 minutes. Spermatozoa in the epididymis were counted by a standard hemocytometric method and motility of sperm (progressive, non- progressive, and immotile) was evaluated under a light microscope (Olympus Optical Co., Japan) at 3 consecutive estimates and reported as mean (20). Sperm viability and morphology were evaluated by the methods described by Turk et al. (21) and Adibmoradi et al. (18). Briefly, a 10 μL sperm suspension was slowly mixed with 40 μL eosinnigrosin (1.67% eosin, 10% nigrosin and 0.1 M sodium citrate). Then, 10 μL of this mixture was transferred to a glass slide and spread slowly by another slide. After preparation of smears, viability and morphology of sperms were evaluated. Spermatozoa with red head were classified as dead sperm and spermatozoa with white head were classified as live sperm (18). Also, sperms were screened and classified into normal and abnormal types, and then the percentage of abnormality was determined for each group (21).

### Tissue preparation for oxidant/antioxidant markers assay

Here, 100 mg of the right testicular tissue sample was homogenized in 500 μL RIPA lysis buffer (1 mM EDTA, 150 mM NaCl, 0.1% sodium dodecyl sulfate (SDS), 1% Triton X-100, 10 mM Tris-HCL; pH=8, 1 mM NaF, 1 mM phenylmethylsulfonyl fluoride) by a glass homogenizer (Heidolph, Germany). Homogenate was centrifuged at 10000 rpm for 15 minutes at 4°C (Centrifuge 5415 R; Eppendorf AG, Germany) and the supernatant was collected and stored at -70°C for subsequent analysis. The protein concentration of the supernatant was estimated using the Bradford method (22). 

### Analyses of lipid peroxidation levels and antioxidant enzymes activities

The content of malondialdehyde (MDA) in the testis was assessed as a lipid peroxidation marker using the thiobarbituric acid reactive substance (TBARS) assay with slight modifications (23). The MDA concentration was obtained based on MDA-TBARS complex optical density at 532 nm wavelength in comparison with the standard curve of MDA. The MDA results are expressed as nmol/mg of protein. Superoxide dismutase (SOD) activity was determined by the nitro blue tetrazolium (NBT) reduction assay, as described by Kakkar et al. (24). Finally, glutathione-peroxidase (GPx) activity was evaluated by a GPx detection kit according to the manufacturer’s instructions (RANSEL, Randox Com, UK). Both SOD and GPx activities are expressed as units/mg protein.

### Testosterone analysis

Testosterone concentration in the serum samples of the experimental groups was quantitatively assessed through enzyme-linked immunosorbent assay (ELISA) using the Diametra testosterone ELISA kit (Diametra Co, Italy), according to the manufacturer’s protocol. Testosterone results are expressed as ng/dl.

### Statistical analysis

Data are expressed as mean ± standard deviation and were analyzed using SPSS 18.0 software (SPSS Inc., Chicago, IL, USA). Differences among various groups were assessed by one-way analysis of variance (ANOVA) followed by the Tukey test. In all cases, P<0.05 was regarded as significant. 

## Results

### Phytochemical content of *A. vera *gel

The results showed that concentrations of the total phenol and flavonoid contents in the AL gel were 49.81 μg GAE/mg and 56.42 μg QE /mg of gel powder, respectively

### Relative weight and dimensions of the testis

The results showed that CIS caused a significant (P<0.05) decrease in relative weight, length, diameter and volume of both the right and left testes compared to the control group. The co-administration of AL and CIS significantly increased relative weight, length and diameter of the testes (right and left), and volume of the right testis compared to the CIS group (P<0.05). Although, there was a numerical increase in the volume of the left testis in the CIS-AL group, it was not statistically significant. Treatment of CIS-treated rats with MET significantly attenuated the reduction of relative weight, length and volume of both the right and left testes and diameter of the left testis (P<0.05). Also, MET increased the diameter of the right testis, however this change was not significant compared to the CIS group (Table 1).

**Table 1:** Relative weight, volume and dimensions of testis in different groups


Groups tes‌ticular parameters	Control	CIS	AL	MET	CIS-AL	CIS-MET

Relative tes‌ticular weight (%)						
Right	0.60 ± 0.03	0.46 ± 0.06^a^	0.61 ± 0.03^b^	0.61 ± 0.04^b^	0.59 ± 0.07^b^	0.60 ± 0.03^b^
Left	0.59 ± 0.02	0.44 ± 0.06^a^	0.62 ± 0.03^b^	0.63 ± 0.06^b^	0.59 ± 0.01^b^	0.59 ± 0.02^b^
Length (mm)						
Right	19.00 ± 2.00	14.00 ± 1.58^a^	19.33 ± 2.08^b^	19.00 ± 1.87^b^	18.60 ± 2.19^b^	18.20 ± 2.86^b^
Left	19.33 ± 0.57	14.50 ± 0.50^a^	19.66 ± 2.08^b^	19.33 ± 1.57^b^	18.20 ± 0.43^b^	18.66 ± 1.15^b^
Diameter (mm)						
Right	8.20 ± 1.30	5.20 ± 0.83^a^	8.33 ± 0.57^b^	8.00 ± 1.00^b^	7.60 ± 1.51^b^	7.40 ± 1.34
Left	8.66 ± 0.57	6.83 ± 0.28^a^	8.66 ± 0.28^b^	8.50 ± 0.50^b^	8.16 ± 0.28^b^	8.33 ± 0.57^b^
Volume (ml)						
Right	1.66 ± 0.15	1.03 ± 0.20^a^	1.73 ± 0.15^b^	1.60 ± 0.10^b^	1.53 ± 0.15^b^	1.50 ± 0.10^b^
Left	1.53 ± 0.23	0.82 ± 0.10^a^	1.53 ± 0.12^b^	1.43 ± 0.15^b^	1.16 ± 0.05	1.23 ± 0.06^b^


Data were expressed as mean ± SD. Values with different superscripts are significantly different: ^a^ ; Significant change from the control group at P<0.05, ^b^; Significant change from the CIS group at P<0.05, Control; Control group, CIS; Cisplatin (5 mg/kg), AL;* A. vera* (400 mg/kg), and MET; Metformin (200 mg/kg).

**Fig.1:**
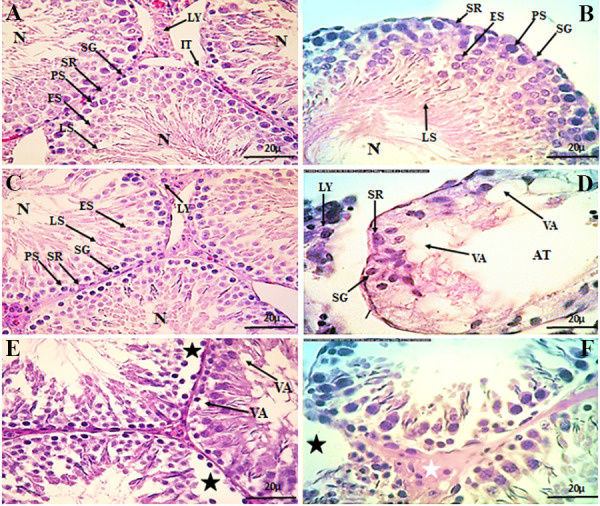
Testicular histopathology in different groups (H&E). **A-C.** The seminiferous tubules with normal germinal epithelium (N), interstitial tissue (IT) and active spermatogenesis in control,* A. vera* (400 mg/kg) and metformin (200 mg/kg) groups, respectively. **D. **Atrophy (AT), vacoulation (VA) and decreasing of spermatogenesisin cisplatin (5 mg/kg) group. **E, F. **A considerable improve in the seminiferous tubules observed in CIS-AL (400 mg/kg* A. vera*+5 mg/kg cispaltin) and CIS-MT (200 mg/kg metformin+5 mg/kg cispaltin) groups, but VA, desquamation (black stars) and interstitial edema (white stars) were seen still. LY; Leydig cell, SR; Sertoli cell, SG; Spermatogonia, PS; Primary spermatocyte, ES; Early spermatid, and LS; Late spermatids.

### Histological findings

The testicular tissue of the control group composed of a high density of normal shape testicular tubules surrounded by interstitial connective tissues. Seminiferous tubules lined by a stratified germinal epithelium, showed features of active spermatogenesis. Spermatogonia cells with heterochromatin and rounded nuclei rested on the basal lamina. Primary spermatocytes were the largest spermatogenic cells in the germinal epithelium with different shapes of chromatin. Furthermore, early-stage spermatids with euchromatin and round nuclei and late-stage spermatids with heterochromatin and elongated nuclei, were attached to the membrane of Sertoli cells. Also, Sertoli cells rested on the basal lamina and had large, euchromatin nuclei with prominent nucleolus. The Leydig cells in interstitial connective tissues had eosinophilic cytoplasm with large and round nuclei (Fig .1A). In AL (Fig .1B, AL) and MET (Fig .1C, MET) treated groups, the seminiferous tubules showed normal cells associations without any structural changes compared to the control group (Fig.1B, C). CIS caused atypical morphological features such as disorganization, and desquamation in the seminiferous tubules. Also, widespread atrophy and loss of all germ cells and extensive vacuolation in the epithelium were observed in CIS-treated rats. In addition, maturation arrest and absence of spermatozoa in the lumen in a majority of seminiferous tubules were significant (Fig .1D). Co-administration of AL and CIS normalized these histological changes and amended spermatogenesis when compared with the CIS alone group; though a slight vacuolation was found, desquamation was still observed in the seminiferous tubules (Fig .1E). Likewise, MET attenuated the histological abnormalities induced by CIS, and protected the testicular tubules although it was less than that seen for AL (Fig .1F).

### Morphometrical finding

**Fig.2:**
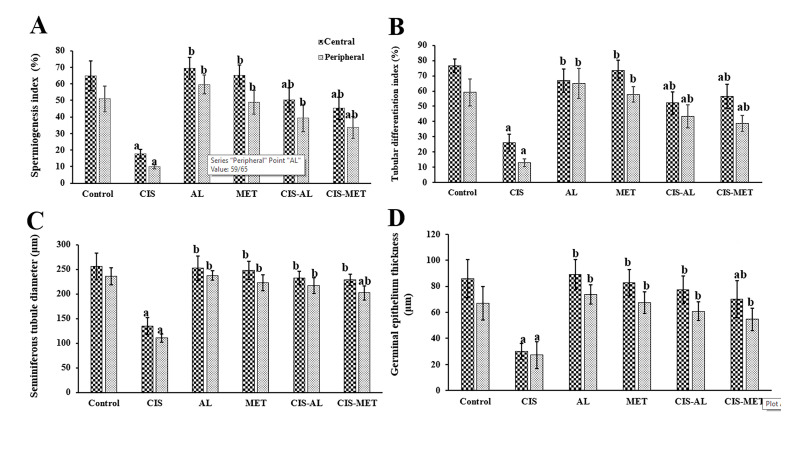
Comparison of the spermatogenesis indexes, germinal epithelium thickness and diameter of seminiferous tubules in different groups. **A.** Spermiogenesis index, **B.** Tubular differentiation index, **C. **Seminiferous tubule diameter, and **D.** Germinal epithelium thickness. Data were expressed as mean ± SD. Values with different superscripts are significantly different: ^a^ ; Significant change from the control group at P<0.05, ^b^ ; Significant change from the CIS group at P<0.05, Control; Control group, CIS; Cisplatin (5 mg/ kg), AL;* A. vera* (400 mg/kg), and MET; Metformin (200 mg/kg).

The number of Sertoli, Leydig, spermatogonia, primary spermatocytes, early and late spermatids cells (Table 2), germinal epithelium thickness, diameter of seminiferous tubule and the spermatogenesis indexes (TDI and SI) decreased in the central and peripheral (sub-capsular) zones of the testis after the CIS treatment (P<0.05, Fig .2). But, administration of AL and MET along with CIS significantly restored these alterations (P<0.05, Table 2, Fig .2). The morphometrical parameters in control, AL, and MET groups were almost identical. 

### Comparison of MDA level and antioxidant activities

**Fig.3:**
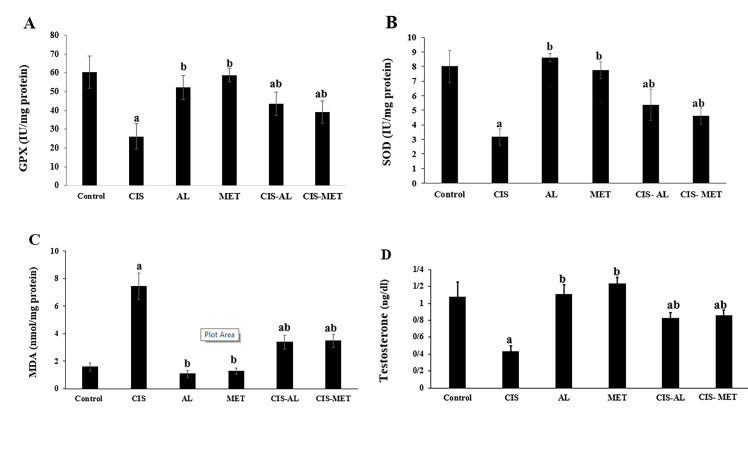
Comparison of antioxidant enzyme activities, malondialdehyde (MDA) levels and serum testosterone levels between groups. **A.** Glutathione peroxidase (GPx) activity, **B. **Superoxide dismutase (SOD) activity, **C.** Malondialdehyde levels, and **D.** Testosterone levels. Data were expressed as mean ± SD. Values with different superscripts are significantly different: ^a^ ; Significant change from the control group at P<0.05, ^b^ ; Significant change from the CIS group at P<0.05, Control; Control group, CIS; Cisplatin (5 mg/kg), AL;* A. vera* (400 mg/kg), and MET; Metformin (200 mg/kg).

Figure 3 shows the changes in MDA level and activities of antioxidant enzymes in testis tissues of different groups. There was a significant increase in MDA level along with a significant reduction of SOD and GPx activities in CIS-treated rats compared to the control group (P<0.05, Fig .3). Nevertheless, administration of AL or MET together with CIS significantly reduced the MDA level and elevated antioxidant enzymes activities in comparison to the CIS group (P<0.05). There was no significant difference in MDA level and antioxidant enzymes activities in the AL and MET groups compared to the control group (Fig .3). 

### Comparison of serum testosterone level

As showed in Figure 3, testosterone level was significantly lower in CIS group rats compared to the other groups (P<0.05, Fig .3). Treatment of CIS rats by AL and MET significantly ameliorated the reduction of testosterone level (P<0.05). AL and MET groups presented no significant difference in the serum testosterone level compared to the control group (P>0.05, Fig .3). 

**Table 2:** Comparison of the number of testicular cells (Leydig, Sertoli, spermatogonia, primary spermatocyte, early and late spermatid) in different groups


Groups parameters	Control	CIS	AL	MET	CIS-AL	CIS-MET

Leydig cells						
Central	16.26 ± 1.85	7.66 ± 1.77^a^	16.01 ± 0.58^b^	15.66 ± 0.61b	12.20 ± 1.00	11.93 ± 1.51
Peripheral	11.26 ± 1.00	5.00 ± 2.16^a^	11.46 ± 0.94^b^	13.66 ± 0.64b	10.86 ± 0.98^b^	10.53 ± 1.36^b^
Sertoli cells						
Central	5.40 ± 0.60	2.33 ± 0.80^a^	5.20 ± 0.91^b^	5.06 ± 0.80b	4.20 ± 0.60	4.40 ± 0.52^b^
Peripheral	5.00 ± 0.91	1.86 ± 0.70^a^	4.60 ± 0.91^b^	5.13 ± 0.64b	3.53 ± 0.11^b^	3.13 ± 0.80
Spermatogonia						
Central	14.46 ± 2.40	6.40 ± 1.50^a^	14.60 ± 0.80^b^	15.33 ± 0.80b	10.80 ± 0.60^b^	10.20 ± 1.24^a^^b^
Peripheral	9.86 ± 2.10	5.86 ± 0.75^a^	10.06 ± 2.20^b^	9.93 ± 1.70b	7.40 ± 1.24^a^^b^	8.13 ± 0.70^a^^b^
Primary spermatocyte						
Central	15.60 ± 0.72	7.26 ± 0.61^a^	15.33 ± 1.40^b^	16.26 ± 1.00b	10.53 ± 0.83^a^^b^	11.00 ± 1.96 ^a^^b^
sub-capsular	7.80 ± 0.60	4.80 ± 0.40^a^	7.33 ± 0.50^b^	7.86 ± 0.41b	6.80 ± 0.40^b^	5.86 ± 0.50^a^
Early spermatid						
Central	63.73 ± 10.21	14.06 ± 2.91^a^	54.63 ± 8.80^b^	58.46 ± 5.28b	38.60 ± 7.68^a^^b^	42.13 ± 3.55^a^^b^
Peripheral	27.13 ± 2.38	7.93±2.60^a^	26.73 ± 1.50^b^	25.40 ± 4.49b	15.26 ± 2.60^a^^b^	17.80 ± 1.40^a^^b^
Late spermatid						
Central	56.66 ± 9.16	19.20±6.39^a^	62.00 ± 8.19^b^	50.46 ± 2.66b	39.80 ± 2.82^b^	44.73 ± 8.76^b^
Peripheral	24.33 ± 3.62	5.66±2.93^a^	26.20 ± 4.72^b^	22.40 ± 2.90b	14.66 ± 3.84	16.06 ± 1.40^b^


Data were expressed as mean ± SD. Values with different superscripts are significantly different: ^a^ ; Significant change from the control group at P<0.05, ^b^; Significant change from the CIS group at P<0.05, Control; Control group, CIS; Cisplatin (5 mg/kg), AL;* A. vera* (400 mg/kg), MET; Metformin (200 mg/kg), CIS-AL;* A. vera* (400 mg/kg)+cisplatin (5 mg/kg), and CIS-MET; Metformin (200 mg/kg)+cisplatin (5 mg/kg).

**Table 3:** Comparison of sperm parameters in different groups


Groups Sperm parameters	Control	CIS	AL	MET	CIS-AL	CIS-MET

Viability (%)	86.26 ± 13.44	30.00 ± 11.33^a^	91.94 ± 11.41^b^	87.00 ± 16.00^b^	77.53 ± 13.66^b^	67.33 ± 9.01^b^
Concentration (10^6^/mL)	120.55 ± 9.44	55.00 ± 7.12^a^	127.50 ± 10.68^b^	128.75 ± 8.75^b^	98.81 ± 13.68^b^	79.75 ± 12.28^a^^b^
Progressive motility (%)	78.17 ± 7.45	11.88 ± 4.16^a^	81.16 ± 7.56^b^	79.69 ± 3.59^b^	64.77 ± 11.78^b^	52.89 ± 17.18^a^^b^
Non- progressive motility (%)	5.76 ± 2.61	15.29 ± 3.90^a^	5.94 ± 3.16^b^	4.58 ± 1.39^b^	7.52 ± 3.72^b^	9.44 ± 3.77
Immotile sperm (%)	16.07 ± 4.99	72.83 ± 5.51^a^	12.90 ± 4.71^b^	15.73 ± 2.20^b^	27.71 ± 8.30^b^	37.67 ± 13.59^a^^b^
Abnormal sperm (%)	8.52 ± 2.40	33.93 ± 3.18^a^	9.65 ± 6.76^b^	11.56 ± 7.38^b^	18.67 ± 4.82^b^	21.64 ± 2.56^a^^b^


Data were expressed as mean ± SD. Values with different superscripts are significantly different:^ a^ ; Significant change from the control group at P<0.05,^ b^; Significant change from the CIS group at P<0.05, Control; Control group, CIS; Cisplatin (5 mg/kg), AL;* A. vera* (400 mg/kg), and MET; Metformin (200 mg/kg).

### Comparison of sperm parameters

A comparison of the groups with regard to sperm parameters is presented in Table 3. The sperm concentration, viability, and progressive and non- progressive motility decreased significantly in the CIS group compared to the control group but the percentage of abnormal sperm morphology increased (P<0.05, Table 3). By contrast, administration of AL gel along with CIS could significantly improve the sperm parameters compared to the CIS group (P<0.05, Table 3). Also, treatment of CIS rats by MET significantly increased sperm concentration, viability and progressive motility and reduced abnormal morphology of sperm compared to the CIS group (P<0.05). Non- progressive motility increased in the CIS-MET group, but this change was not significant compared to the CIS group (Table 3).

No significant differences were observed in the sperm parameters between the AL and MET groups and the control group (Table 3).

## Discussion

CIS-based chemotherapy induces gonadal toxicity and infertility by increasing oxidative stress (5, 6). Hence, administration of antioxidant agents may be a useful strategy in reducing CIS toxicity and preserve the fertilization capacity of patients receiving CIS.

The results of the present study showed that CIS decreased relative weight and dimensions of the testis, and reduced the germinal epithelium thickness, and the diameter of seminiferous tubules. Additionally, histopathological changes such as testicular atrophy, desquamation, vacuolation of germinal epithelium, and reduction of spermatogenesis activity were observed in CIS-treated rats.

Loss of testicular weight and dimension in CIS-treated rats could be due to the inhibition of spermatogenesis, atrophy of testicular tubules, reduction of spermatogenic cells, and other degenerative alterations caused by CIS (25). These histological damages may be explained by disruptions of the redox balance induced by CIS which result in DNA damage, lipid peroxidation, and inhibition of protein synthesis (4). Testis tissue is highly vulnerable to oxidative stress because it has a high metabolic activity and considerable amount of highly unsaturated fatty acids (26). Free radicals impair different parts of the testis especially testicular germinal cells and lead to atrophy in testicular tubules and reduction of sperm generation (20, 26, 27).

Data from the present study likely showed that CIS treatment impairs oxidant-antioxidant balance in testicular tissue so that it increased the levels of MDA and decreased antioxidant enzymes (SOD and GPx) activities, these results are in agreement with previous reports (27, 28). The peroxidation of lipids is one of the toxic effects of CIS in the testis and MDA is produced as the end-product of this process; thus, MDA content is the best marker for measuring oxidative stress and lipid peroxidation indirectly. Also, the increase in the MDA level may be related to DNA fragmentation as reported previously (29). The reductions of the antioxidant enzymes activities observed in this study, are probably due to either direct effects of CIS on these enzymes or enhanced consumption of antioxidant enzymes for detoxifying free radicals generated by CIS (30).

We found a CIS-mediated decrease in serum testosterone concentration which is fundamentally consistent with previous studies (27, 29). Saral et al. (27) reported that the reduction of testosterone level induced by CIS results from a decrease in the number of Leydig cells or their dysfunction. Another hypothesis is that CIS inhibits testosterone synthesis by depressing the cytochrome P‐450‐dependent 17‐α‐hydroxylase level and decreasing the numbers of luteinizing hormone (LH) receptors in Leydig cells.

CIS treatment reduced sperm concentration, motility and viability and increased abnormal sperm morphology, consistent with many reports that have indicated the side effects of CIS on sperm function (20, 28). The alteration in sperm parameters of the CIS group was probably caused by prolonged exposure of the testis to CIS-induced free radicals (20). Free radicals decrease the mitochondrial membrane potential in sperm cells which is associated with a decrease in adenosine triphosphate (ATP) production and inhibition of sperm motility (31). In addition, damage of the sperm cell membrane by CIS-induced free radicals may be the cause for the decrease in sperm viability and motility and the increase in the morphological defects (32).

In the present study, administration of AL gel at a dose of 400 mg/kg effectively inhibited the CIS-induced testicular oxidative stress by decreasing the MDA levels and increasing the antioxidant enzymes activities. Also, our results clearly showed that AL treatment attenuated adverse effects of CIS on relative testicular weight and dimension, sperm parameters, testosterone level, and histological changes of the testis. 

These chemoprotective effects of AL a gainst CIS-induced toxicity may be related to the antioxidant effect of AL, as reported in previous studies (33, 34). Imaga et al. (33) reported that AL gel improves CIS-induced oxidative damages in the kidney and liver of experimental animals. Also, Chatterjee et al. (34) indicated that administration of AL along with CIS was associated with amelioration of antioxidant defense system and diminution of CIS-induced nephrotoxicity.

AL and especially its gel are highly spermatogenic and enhance male fertility by elevating sperm quality (10, 11). AL increases spermatogenesis process via affecting spermatogenic cells and stimulating cell division, and increases testosterone hormone by stimulating Leydig cells (10, 11, 35).

Estakhr and Javdan (10) reported that AL significantly increased testicular weight, testosterone hormone, and sperm concentration and motility and decreased sperm abnormalities. Also, AL increases cAMP responsive element modulator (CREM) gene expression that has a key role in the regulation of the expression of genes that control spermatogenesis (11). 

AL contains a large number of antioxidant compounds including vitamins (A, C, B, E), flavonoids, phenolic compounds, and polysaccharides (7). Vitamin E has the highest antioxidant activity and plays a key role in the protection of plasma membrane against peroxidation by free radicals. Also, vitamin E improves testicular weight, germinal epithelium thickness, and diameter size of seminiferous tubule (36). Vitamin C in AL gel performs an important role in the integrity and fertility of semen and makes up to 65% of the total antioxidant capacity of seminal plasma. Also, vitamin C inhibits sperm agglutination and increases testosterone concentration (37). Furthermore, phenolic compounds and polysaccharides of AL have antioxidant capacity and prevent diseases induced by oxidative stress (38). Therefore, because of its antioxidant properties, AL can reduce CIS-induced oxidative damages in testis tissue and can support spermatogenesis and protect spermatozoa against free radicals. 

## Conclusion

Our findings demonstrated that oxidative stress can play a significant role in the pathogenesis of CIS-induced testicular and sperm injuries. Also, biochemical, hormonal, and histological results suggest that AL gel could be effective for prevention of gonadal toxicity induced by CIS in male Wistar rats. This study concluded that AL gel due to its potent antioxidant effect, can protect the testicular tissue from toxic damages caused by CIS.
